# Minimal shoes improve stability and mobility in persons with a history of falls

**DOI:** 10.1038/s41598-020-78862-6

**Published:** 2020-12-10

**Authors:** Tomasz Cudejko, James Gardiner, Asangaedem Akpan, Kristiaan D’Août

**Affiliations:** 1 Department of Musculoskeletal and Ageing Science, Institute of Life Course and Medical Sciences, University of Liverpool, Liverpool, UK; 2 Department of Medicine for Older People and Stroke, Liverpool University Hospitals NHS Foundation Trust, Liverpool, UK

**Keywords:** Geriatrics, Lifestyle modification, Rehabilitation

## Abstract

Postural and walking instabilities contribute to falls in older adults. Given that shoes affect human locomotor stability and that visual, cognitive and somatosensory systems deteriorate during aging, we aimed to: (1) compare the effects of footwear type on stability and mobility in persons with a history of falls, and (2) determine whether the effect of footwear type on stability is altered by the absence of visual input or by an additional cognitive load. Thirty participants performed standing and walking trials in three footwear conditions, i.e. conventional shoes, minimal shoes, and barefoot. The outcomes were: (1) postural stability (movement of the center of pressure during eyes open/closed), (2) walking stability (Margin of Stability during normal/dual-task walking), (3) mobility (the Timed Up and Go test and the Star Excursion Balance test), and (4) perceptions of the shoes (Monitor Orthopaedic Shoes questionnaire). Participants were more stable during standing and walking in minimal shoes than in conventional shoes, independent of visual or walking condition. Minimal shoes were more beneficial for mobility than conventional shoes and barefoot. This study supports the need for longitudinal studies investigating whether minimal footwear is more beneficial for fall prevention in older people than conventional footwear.

## Introduction

Falls occur in 30–60% of older adults each year, and 10–20% of these result in injury, hospitalisation and/or death^1^. Costs resulting from falls in 2009 alone ranged between 0.85 and 1.5% of the total healthcare expenditure within the United States of America and the European Union^2^. The prevalence and impact of falls are likely to carry on increasing because of the expected rise in ageing populations. Strategies to prevent falls in older adults are therefore crucial and timely.

Postural and walking instabilities have been recognized as major risk factors for frequent falls in older adults^1^. Furthermore, it is well accepted that plantar sensation from the cutaneous receptors, is a critical element to stability during standing and walking^3–5^. Although the causes of falls are complex and multifactorial, shoes alter gait and impair control of stability via diminished perception of walking surfaces^6–8^, which may result in an increased risk of falling^9,10^. For these reasons, studies investigating the effect of footwear styles on postural and walking stability in older adults are necessary—and research is lacking in this area as indicated by systematic reviews on the topic^10,11^.

Cushioning, arch supports, restrictive toe boxes and raised heels are all features of modern conventional footwear (Supplementary Fig. S1A/B). Highly structured and supportive shoes may limit the sensory input to the brain and affect the control of gait, as the foot is not as susceptible to changes in shape, pressure and touch as walking barefoot^12^. Although it is not common to walk barefoot in a modern society, it is reasonable to suggest that the use of a shoe that can reproduce the neuro-mechanical properties of barefoot gait may help minimize the risk of falls in older people. We have previously compared multiple prototypes of a minimal shoe (Supplementary Fig. S1C) with barefoot and conventional shoes in middle-aged and older people^13^. We showed that independent of the design features, wearing minimal shoes was not different than barefoot for postural and walking stability, but was more beneficial than wearing conventional shoes. We also observed that prototypes with wider and harder soles were more beneficial for stability and mobility than prototypes with narrower and softer soles^13^.

Despite initial promising results^14^, studies evaluating the effects of minimal shoes in older adults are limited. To our knowledge, there are no studies comparing the effects of conventional shoes, minimal shoes and barefoot on stability and mobility in persons with a history of falls. In addition, given that visual, vestibular and cognitive systems deteriorate during ageing^15^, determining the effects of footwear type on postural and walking stability in the absence of visual input and/or during an additional cognitive load are crucial, and research is particularly lacking in this area. Finally, to be regarded a practical intervention, minimal footwear needs to be acceptable to older adults from the perspective of comfort, ease of use and aesthetics^16^, and this has not been evaluated before in comparison to conventional shoes.

Therefore, building on the results of our previous research^13^, we here test the performance of the market-available minimal shoes by comparing its effects on stability, mobility, and design perceptions with conventional shoes and barefoot among persons with a history of falls. We hypothesise that wearing minimal shoes will be more beneficial for stability than wearing conventional shoes.

## Results

### Participants’ characteristics

Thirty persons with a history of falls participated in the study. They had a mean age of 68.6 ± 4.4 years, a mean height of 1.68 ± 0.08 m, a mean weight of 75.5 ± 13.6 kg, a mean BMI of 26.6 ± 3.7 kg/m^2^, and 17 (57.0%) were female.

### Postural stability

Results of the linear mixed effect model, examining the effect of footwear (conventional vs. minimal vs. barefoot) and visual condition (eyes open vs. eyes closed), revealed main effects of footwear on all four metrics of postural stability (all *p*’s < 0.050) and a main effect of visual condition on anterior–posterior (AP) velocity of the center of pressure (CoP) (*p* < 0.001), but not on the remaining three metrics. Specifically, we observed significantly lower values of CoP metrics (better postural stability) when wearing minimal shoes and when being barefoot compared to wearing conventional shoes (all *p*’s < 0.050). There was no significant difference between minimal shoes and barefoot. There was no significant interaction between footwear and visual condition. Full results of the statistical analyses are presented in the Table 1. Means and SDs for the CoP metrics of postural stability are presented in the Supplementary Table S1.

**Table 1:** Effects of footwear (CV vs. ML vs. BF), visual condition (EO vs. EC) and footwear * visual condition on the CoP metrics of postural stability (n = 30).

CoP metric	Parameter	Estimate	*SE*	*df*	Sig	95% CI lower bound	95% CI upper bound
*AP velocity*	Footwear (CV vs. ML)	0.143	0.034	180	**< 0.001**	0.076	0.211
	Footwear (BF vs. ML)	− 0.045	0.034	180	0.187	− 0.112	0.022
	Footwear (BF vs. CV)	− 0.174	0.023	180	**< 0.001**	− 0.235	− 0.113
	Standing condition (EO vs. EC)	− 0.130	0.034	180	**< 0.001**	− 0.197	− 0.062
	Footwear (CV vs. ML) * Visual	− 0.008	0.048	180	0.856	− 0.104	0.086
	Footwear (BF vs. ML) * Visual	0.014	0.048	180	0.758	− 0.080	0.110
	Footwear (BF vs. CV) * Visual	0.014	0.047	180	0.679	− 0.086	0.106
*AP rom*	Footwear (CV vs. ML)	0.100	0.039	180	**0.013**	0.021	0.178
	Footwear (BF vs. ML)	− 0.044	0.039	180	0.265	− 0.123	0.034
	Footwear (BF vs. CV)	− 0.126	0.028	180	**< 0.001**	− 0.194	− 0.058
	Standing condition (EO vs. EC)	− 0.072	0.039	180	0.072	− 0.150	0.006
	Footwear (CV vs. ML) * Visual	0.014	0.056	180	0.803	− 0.097	0.125
	Footwear (BF vs. ML) * Visual	0.051	0.056	180	0.360	− 0.059	0.162
	Footwear (BF vs. CV) * Visual	0.043	0.050	180	0.706	− 0.092	0.111
*ML velocity*	Footwear (CV vs. ML)	0.243	0.042	180	**< 0.001**	0.158	0.327
	Footwear (BF vs. ML)	− 0.012	0.042	180	0.775	− 0.096	0.071
	Footwear (BF vs. CV)	− 0.213	0.030	180	**< 0.001**	− 0.286	− 0.141
	Standing condition (EO vs. EC)	− 0.047	0.042	180	0.266	− 0.131	0.036
	Footwear (CV vs. ML) * Visual	− 0.044	0.060	180	0.461	− 0.163	0.074
	Footwear (BF vs. ML) * Visual	0.039	0.060	180	0.517	− 0.079	0.158
	Footwear (BF vs. CV) * Visual	0.015	0.052	180	0.805	− 0.095	0.126
*ML rom*	Footwear (CV vs. ML)	0.184	0.049	180	**< 0.001**	0.086	0.283
	Footwear (BF vs. ML)	− 0.026	0.049	180	0.595	− 0.124	0.071
	Footwear (BF vs. CV)	− 0.186	0.035	180	**< 0.001**	− 0.271	− 0.101
	Standing condition (EO vs. EC)	− 0.010	0.049	180	0.831	− 0.108	0.087
	Footwear (CV vs. ML) * Visual	− 0.010	0.070	180	0.881	− 0.149	0.128
	Footwear (BF vs. ML) * Visual	0.040	0.070	180	0.567	− 0.099	0.179
	Footwear (BF vs. CV) * Visual	0.036	0.056	180	0.707	− 0.093	0.131

*CV* conventional shoes, *ML* minimal shoes, *BF* barefoot, *EO* eyes open, *EC* eyes closed, *CoP* center of pressure, *AP* anterior–posterior, *ML* medio-lateral, *rom* range of motion, *SE* standard error, *df* degree of freedom, sig.- *p* value, *CI* confidence interval.

### Walking stability

**Figure 1:**
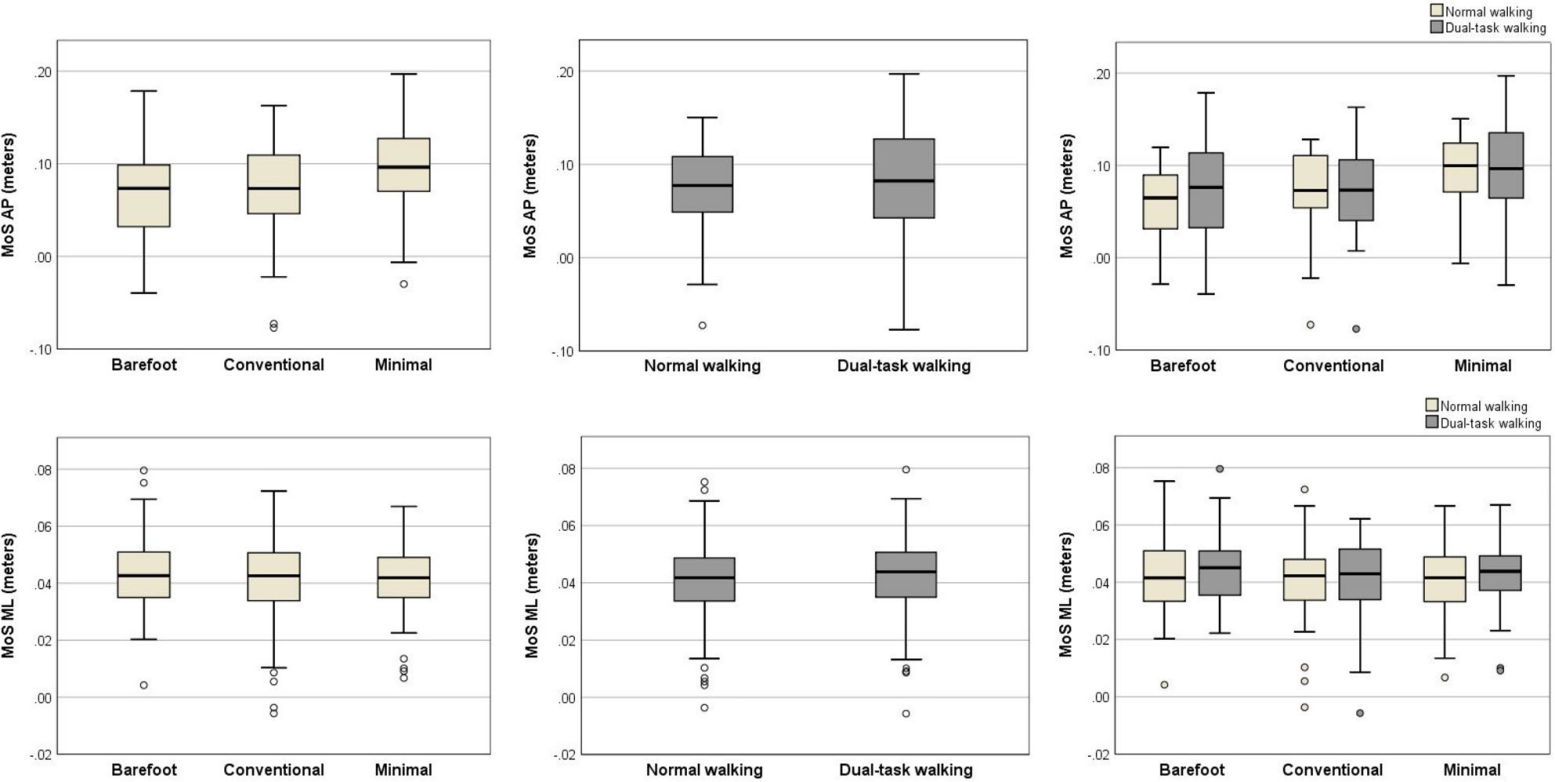
Footwear, walking condition and footwear * walking condition values of the MoS metrics of walking stability. The box represents the median and interquartile range, the whiskers represent the most extreme values within 1.5 times of the interquartile range beyond the 25th and 75th percentile, and the circles represent the more extreme values. *MoS* margin of stability, *AP* anterior–posterior, *ML* medio-lateral.

Results of the linear mixed effect model (adjusted for cadence and/or walking speed) examining the effect of footwear (conventional vs. minimal vs. barefoot) and walking condition (normal walking vs. dual-task walking), revealed main effects of footwear on AP Margin of Stability (MoS) (*p* < 0.050), but not on the medial–lateral (ML) MoS. Specifically, we observed significantly higher value of MoS AP (better walking stability) when walking in minimal shoes compared to walking in conventional shoes (*p* < 0.001) and barefoot (*p* < 0.001). There was no significant effect of walking condition on the MoS AP nor on the MoS ML. There was no significant interaction between footwear and walking condition. Full results of the statistical analyses are presented in the Table 2. Medians and interquartile ranges of MoS metrics of walking stability are presented in the Fig. 1.

**Table 2:** Effects of footwear (CV vs. ML vs. BF), walking condition (NW vs. DTW) and footwear * walking condition on the MoS metrics of walking stability (n = 28).

MoS metric	Parameter	Estimate	*SE*	*df*	Sig	95% CI lower bound	95% CI upper bound
*MoS AP*	Footwear (CV vs. ML)	− 0.020	0.002	136	**< 0.001**	− 0.026	− 0.014
	Footwear (BF vs. ML)	− 0.027	0.003	140	**< 0.001**	− 0.034	− 0.020
	Footwear (BF vs. CV)	− 0.007	0.004	149	0.163	− 0.002	0.015
	Walking condition (NW vs. DTW)	0.001	0.002	133	0.899	− 0.006	0.007
	Footwear (CV vs. ML) * Walking	0.002	0.004	126	0.521	− 0.005	0.011
	Footwear (BF vs. ML) * Walking	− 0.003	0.004	126	0.390	− 0.013	0.005
	Footwear (BF vs. CV) * Walking	− 0.002	0.003	126	0.478	− 0.012	0.006
	Speed	− 0.356	0.021	148	**< 0.001**	− 0.398	− 0.315
	Cadence	− 0.003	0.001	152	**< 0.001**	− 0.004	− 0.002
*MoS ML*	Footwear (CV vs. ML)	− 0.001	0.001	140	0.254	− 0.005	0.001
	Footwear (BF vs. ML)	0.001	0.001	142	0.319	− 0.001	0.004
	Footwear (BF vs CV)	0.003	0.001	148	0.134	− 0.001	0.006
	Walking condition (NW vs. DTW)	− 0.001	0.001	140	0.486	− 0.004	0.002
	Footwear (CV vs. ML) * Walking	0.001	0.002	140	0.526	− 0.003	0.005
	Footwear (BF vs. ML) * Walking	− 0.001	0.002	140	0.846	− 0.004	0.004
	Footwear (BF vs. CV) * Walking	− 0.001	0.002	140	0.788	− 0.004	0.005
	Speed	− 0.0169	0.007	167	**0.033**	− 0.032	− 0.001

*CV* conventional shoes, *ML* minimal shoes, *BF* barefoot, *NW* normal walking, *DTW* dual-task walking, *MoS* margin of stability, *AP* anterior–posterior, *ML* medio-lateral, *SE* standard error, *df* degree of freedom, *sig. p* value, *CI* confidence interval.

### Mobility

The linear mixed effect model revealed main effects of footwear on the Timed up and Go (TUG) test and the modified Star Excursion Balance (SEB) test in all directions (all *p*’s < 0.050). Specifically, we observed significantly lower time to complete the TUG test (better mobility), when wearing minimal shoes compared to when being barefoot (*p* = 0.001) and when wearing conventional shoes (*p* = 0.001). In regards to the SEB test, participants had a greater reach distance (better mobility) in anterior, posterior, lateral and medial directions, when wearing minimal shoes than when wearing the conventional shoes and when being barefoot (all *p*’s < 0.050). Full results of the statistical analyses are presented in the Table 3. Means and SDs for the TUG test and the SEB test are presented in the Supplementary Table S3.

**Table 3:** Effects of footwear (CV vs. ML vs. BF) on the TUG test (n = 30) and the modified SEB test (n = 25).

Mobility	Parameter	Estimate	*SE*	*df*	Sig	95% CI lower bound	95% CI upper bound
*TUGT*	Footwear (CV vs. ML)	0.298	0.073	60	**0.001**	0.112	0.483
	Footwear (BF vs. ML)	0.340	0.081	60	**0.001**	0.135	0.546
	Footwear (BF vs. CV)	0.043	0.083	60	1.000	− 0.169	0.255
*SEBT A*	Footwear (CV vs. ML)	− 1.220	0.382	50	**0.012**	− 2.204	− 0.236
	Footwear (BF vs. ML)	− 1.097	0.326	50	**0.008**	− 1.937	− 0.258
	Footwear (BF vs. CV)	0.123	0.587	50	1.000	− 1.388	1.633
*SEBT P*	Footwear (CV vs. ML)	− 4.244	1.339	50	**0.012**	− 7.689	− 0.799
	Footwear (BF vs. ML)	− 4.392	1.136	50	**0.002**	− 7.311	− 1.466
	Footwear (BF vs. CV)	− 0.148	0.867	50	1.000	− 2.378	2.082
*SEBT L*	Footwear (CV vs. ML)	− 2.659	0.497	50	**< 0.001**	− 3.939	− 1.378
	Footwear (BF vs. ML)	− 2.839	0.923	50	**0.016**	− 5.214	− 0.463
	Footwear (BF vs. CV)	− 0.180	0.614	50	1.000	− 1.760	1.400
*SEBT M*	Footwear (CV vs. ML)	− 3.548	0.787	50	**< 0.001**	− 5.573	− 1.523
	Footwear (BF vs. ML)	− 2.008	0.734	50	**0.035**	− 3.897	− 0.119
	Footwear (BF vs. CV)	1.540	0.574	50	**0.039**	− 3.017	− 0.063

*CV* conventional shoes, *ML* minimal shoes, *BF* barefoot, *TUGT* timed up and go test, *SEBT* star excursion balance test, *A* anterior, *P* posterior, *L* Lateral, *M* medial–lateral, *SE* standard error, *df* degree of freedom, *sig. p* value, *CI* confidence interval.

### Perceptions of the shoes

Results of the paired-samples t-test examining differences in perceptions between conventional shoes and minimal shoes revealed that, compared to the conventional shoes, participants perceived the minimal shoes as having a better fit (md 16.0, std. error md 5.1, 95% CI 5.5 26.0, *p* = 0.004), and as being lighter (md − 9.1, std. error md 2.5, 95% CI − 14.2 − 3.9, *p* = 0.001). They also perceived walking in minimal shoes as more stable compared to walking in the conventional shoes (md 21.4, std. error md 5.3, 95% CI 10.5 32.2, *p* < 0.001). There were no significant differences between the shoes in terms of attractiveness, attractiveness for others, comfort, and ease of donning and doffing. Means and SDs for the perceptions of the shoes are presented in the Supplementary Table S4.

## Discussion

To the best of our knowledge, this is the first study comparing the effects of minimal shoes, conventional shoes and barefoot on stability and mobility in persons with a history of falls. It is also the first study investigating whether the effect of footwear type on stability is altered by the absence of visual input or by an additional cognitive load in persons with a history of falls.

**Figure 2:**
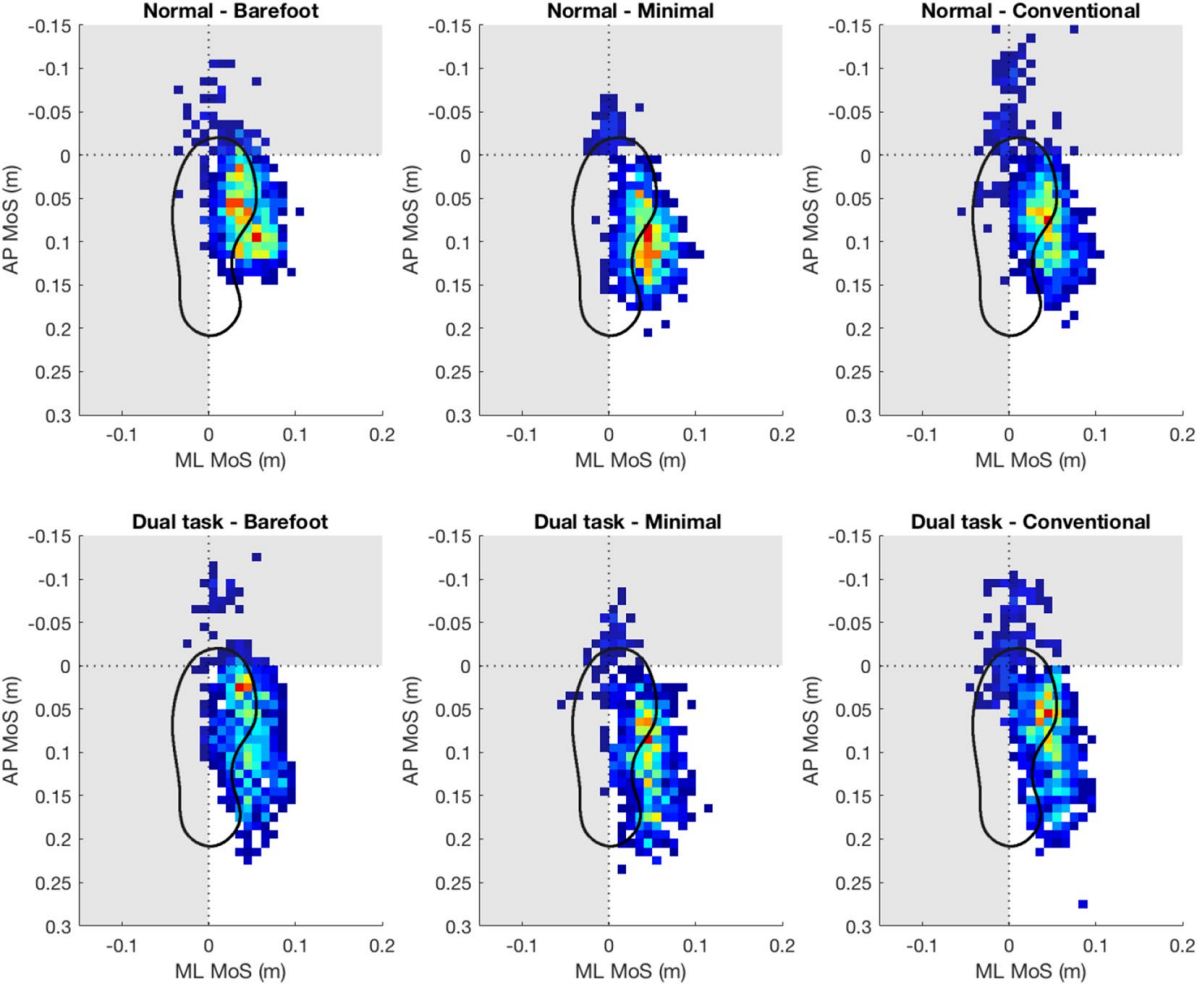
Two-dimensional histogram of extrapolated centre of mass (xCoM) position at heel strike for walking trials. Hotter colours indicate xCoM at heel strike was positioned in that area more frequently. Greyed out areas represent regions considered ‘unstable’. Lower values of the margin of stability (MoS) are indicative of worse walking stability. The boundary of support (dsotted lines) is defined by the calcaneus marker for medio-lateral (ML) direction and hallux for the anterior–posterior (AP) direction. Data are plotted for all steps (left and right foot) from all participants for each condition. Histogram bins are 10 mm squares.

First, we show that wearing minimal shoes and being barefoot is more beneficial for postural stability than wearing conventional shoes (Table 1, Supplementary Table S1). This is in line with our previous study in healthy middle-aged and older people^13^ and extends it to persons with a history of falls. This is in contrast to Broscheid and Zech^17^ who reported negative effects on postural control during barefoot and minimal shoe conditions compared to conventional shoes. This difference might be explained by different footwear and/or methods used in both studies. We assessed postural stability with a pressure plate and quantified it from the movement of the center of pressure. Whereas, Broscheid and Zech used the Balance Error Scoring System, which involves a performance based physical test in which an examiner counts errors, or deviations from the proper stance, accumulated by the subject. We also show that wearing minimal shoes was more beneficial for walking stability in the sagittal plane (MoS AP) than walking in conventional shoes and barefoot (Table 2, Fig. 1) This matches results from a recent study by Petersen et al.^14^ who showed that using minimal shoes was more beneficial for stability during walking than being barefoot in healthy older people, as expressed by increased local dynamic stability and decreased gait variability. Using a different metric of stability, we validate our previous study and extend it by including comparison to conventional shoes in persons with a history of falls, showing these results are robust and not just a feature of methodological approach. Of particular note is that participants also perceived walking in minimal shoes as more stable than walking in conventional shoes (Supplementary Table S4), which adds further support to this observation. We, however, did not observe any differences between footwear conditions for walking stability in the frontal plane (MoS ML). More beneficial effects of minimal shoes on walking stability compared to barefoot and conventional shoes can be seen visually in the Fig. 2. The distribution of the extrapolated center of mass position for minimal shoes is typically more condensed and further away from the anterior–posterior border (horizontal line) and medial–lateral border (vertical line) of the base of support resulting in larger values of the MoS and improved stability during walking compared to barefoot and conventional shoes.

Finally, we demonstrate, that using minimal shoes is more beneficial for mobility in persons with a history of falls than barefoot and conventional shoes. Participants completed the TUG test approximately 0.3 s faster when in minimal shoes compared to barefoot or when wearing conventional shoes (Table 3, Supplementary Table S3), which is comparable to our previous results in healthy middle-aged and older people^13^. In addition, we observe that participants performed better during the SEB test, when in minimal shoes than in barefoot or conventional shoes (difference in reach distance ranged 1–4.4 cm) (Table 3, Supplementary Table S3). The clinical significance of the effects on stability and mobility is unclear and should be viewed in the light of this being a cross-sectional observational study in a laboratory environment, and the short duration of use of the footwear. Although footwear effects on mobility have been studied before in older people^18^, the results seem conflicting and direct comparison are difficult due to the variability of the footwear used. Further research is required to understand if our findings extend to long-term benefits and a reduction in the fall risk for older people.

Several plausible mechanisms explain the differences in stability and mobility between minimal and conventional shoes in the current study. First, heel elevation present in the conventional shoe might have modified the posture and shifted the total body centre of mass anteriorly, closer to the boundary of the base of support^19^. In addition, the curved aspect of the conventional shoes and the potential “rocking effect” could have impaired the role of toe flexors during the control of gait leading to reduced AP MoS^20^. On the other hand, this curved aspect could have resulted in different toe marker placements and thus different ‘foot length’ (i.e. toe-heel marker) between minimal shoes and conventional shoes. However, there was no statistically significant difference in ‘foot length’ between minimal shoes and conventional shoes (md 0.003 m; *p* = 1.000), indicating that difference in foot length cannot explain the observed difference in AP MoS between conventional shoes and minimal shoes. Next, although we observed benefits of minimal shoes over conventional shoes in medio-lateral postural stability, but not in the medio-lateral walking stability, conventional shoes with higher heel height may also lead to lateral instability as they present a higher tipping angle compared to minimal shoes with lower heel height^21,22^. Moreover, our study supports previous literature indicating the importance of a footwear with a wide sole for fall prevention^21^, as these effects might be explained by a larger base of support and enhanced somatosensory information from the skin receptors in the foot sole to the brain. Kennedy and Inglis^23^ observed increased concentration of cutaneous mechanoreceptors in the forefoot of the human sole and its lateral borders. These receptors are sensitive to contact pressures^24^ and may be sensitive to potential changes in the distribution of pressure^4^. The wider sole in the minimal shoes might have allowed the pressures to be distributed more evenly across the foot, potentially leading to stimulation of plantar mechanoreceptors located in the regions that are not normally stimulated in a conventional shoe with a narrower sole. It is plausible that the central nervous system used this additional sensory information to elaborate on descending motor strategies (i.e. improved muscle activity). The thinner aspect of the sole in minimal shoes might also further augment the stimulation of plantar cutaneous mechanoreceptors by increasing the in-shoe pressure. On the other hand, it might make the foot more vulnerable to discomfort or pain on certain surfaces or if an unexpected obstacle is encountered, potentially leading to an increased chance of a fall in an outdoor environment. Although, the calculation of the MoS metrics of walking stability is reliant on walking speed and is sensitive to changes in cadence (via decreased step length)^25^, even after adjusting the statistical models for speed and cadence, the significant differences between minimal shoes and conventional shoes in MoS AP remained—suggesting the presence of other underlying mechanisms which need to be investigated in future research (i.e. lower extremity muscle activity).

Second, we observe that footwear type has an effect on both postural stability and walking stability regardless of visual condition (eyes open vs. closed) or walking condition (normal vs. dual task walking). The stability of posture during standing and walking is maintained by the rapid processing of, mainly, vestibular and visual inputs in the central nervous systems, followed by outputs to the musculoskeletal systems. Every factor in these systems deteriorates during aging. In addition, poor stability and mobility in aging has been associated with exaggerated effects of cognitive-motor dual tasking and cognitive impairment^26^. It is known that the human postural control system relies more heavily on the peripheral somatosensory system, such as this from feet plantar cutaneous receptors, for proprioceptive feedback to maintain stability when visual and cognitive systems are compromised^27^. Thus, we hypothesised that footwear types might have different effects on stability during standing and walking, during experimentally induced absence of visual input and during an additional cognitive load while walking. However, our results do not confirm this hypothesis. The reason behind this might be two-fold. First, the absence of visual input might have not been challenging enough task for postural stability, when stood on both feet. Perhaps when faced with a task more challenging to postural stability, such a standing on only one foot, the influence of reduced visual input would be much more significant. Second, our population was relatively young, therefore, the dual-motor task employed in this study might not have been challenging enough on stability during walking, to require a significant change in kinematics or an increased input from feet mechanoreceptors. Future studies should determine whether the effect of footwear type on stability is altered by the absence of visual input or by an additional cognitive load by recruiting older populations and employing more challenging tasks for postural and walking stability.

This study has some limitations. First, testing participants in new shoes may influence postural and gait responses to footwear. However, we allowed participants sufficient time to become accustomed to the new footwear^28^. Second, our study population (mean age 68 years) might not be representative of the population of fallers at ages > 70. Future studies should test footwear in older groups to add in scope to this study. In addition, we did not collect data on feet sensitivity and lower limb proprioception—which might be potential moderators/mediators of the observed beneficial effects of minimal footwear. We suggest that future studies include such testing in their study protocols. Moreover, we recommend to explore the differences between footwear types during walking subjected to slips and/or trips—both major contributing factors to falls in older people^29^ Finally, the findings should be treated with caution when applied to real-life situations, since the testing was conducted in a safe laboratory environment which might not translate to performance in the activities of everyday life of older people. Longitudinal studies are needed to extend current findings to daily life of older people.

This study shows that wearing minimal shoes is more beneficial for stability and mobility in persons with a history of falls than wearing conventional shoes. This study helps older adults, clinicians who care for them, and shoe designers to make better-informed choices regarding footwear. Given that reduced stability and mobility are one of the key risk factors for falls in older adults, this study supports the need for longitudinal studies investigating whether using minimal shoes is more appropriate for fall prevention that using supportive and cushioned conventional shoes.

## Methods

### Study design

This was a cross-sectional observational study with a within-participant repeated-measures design. Participants undertook assessments in three footwear conditions in a randomised order i.e. (1) conventional shoes, (2) minimal shoes, and (3) barefoot. We used simple randomisation generated from the website www.randomizer.org. Health Research Authority, Health and Care Research Wales, and East Midlands—Derby Research Ethics Committee approved the study (reference 19/EM/0197). The study protocol had been published prior to the inclusion of the first participant (NCT03874728). All methods were performed in accordance with the relevant guidelines and regulations.

### Participants

Persons with a history of falls were recruited from the local community via adverts in GPs, University of Third Age and the University of Liverpool intranet, between November 2019 and March 2020. The inclusion criteria were: age ≥ 60 years, and ≥ 1 self-reported fall after the age of 60^30^. A qualifying fall was defined as an unintentional fall to the ground, not preceded by loss of consciousness and not resulting from an external force (such as being pushed or hit). Exclusion criteria were self-reported: (1) presence of a macro-vascular condition (angina, stroke, peripheral vascular disease or diabetes) or a neuromuscular disease (multiple sclerosis, Alzheimer’s disease or Parkinson’s disease), (2) use of a walking aid (cane or walker), (3) ankle, knee or hip surgery ≤ 3 months, and/or (4) pain of ≥ 8 on the Numeric Rating Scale (0—not pain at all, 10—worst pain imaginable). All participants provided written informed consent according to the Declaration of Helsinki.

### Footwear conditions

#### Conventional shoes

We tested the Go Walk 4.0-Pursuit for females (Skechers USA, Inc.; Supplementary Figure S1A) and the Superior 2.0-Jeveno shoe for males (Skechers USA, Inc.; Supplementary Figure S1B). These models were chosen because they differ from the minimal shoes in terms of sole width, sole thickness and flexibility.

#### Minimal shoes

We tested market-available minimal shoes (Primus Knit, Vivobarefoot Ltd., London, UK; Supplementary Figure S1C) with properties (wider sole and shore hardness OS 75) which most closely match the protype with best performance in our previous research^13^.

#### Barefoot

Participants also undertook all assessments barefoot.

The study coordinator fitted each participant with the shoes by palpating the participant’s hallux during standing to ensure that there was approximately 0.5–2 cm between the hallux and shoe end. To ensure correct fitting, participants were asked if they were comfortable in the shoe and if they felt it was appropriately fitted. To minimise participants’ bias, we did not inform participants about the characteristics of the footwear conditions or the study hypotheses.

### Outcome measures

#### Postural stability

Postural stability was expressed with movement of the centre of pressure (CoP) during standing with eyes open and eyes closed. CoP movement was measured using a pressure plate (FootWork Pro, AM CUBE, Berkshire, UK) with 4,096 sensors, dimensions 490 × 490 × 7.6 mm and a sampling frequency 40 Hz. This sampling frequency is sufficient because the kinematics of the movement we studied (quiet standing) occur at frequencies lower than 20 Hz^31^. Sampling frequency does not appear to significantly affect reliability of the CoP metrics with generally consistent reliabilities (r = 0.82–0.89) reported across different frequencies ranging from 20 to 200 Hz^32,33^. Participants undertook three trials of 30 s while standing still on both feet. Intra-tester and inter-tester reliability for this method have been reported as high in older people^32^. To gain a diverse description of the CoP movement, the selection of CoP metrics should include both distance as well as time–distance based parameters^32^. Thus, postural stability was quantified by computing the mean velocity (mm/s) and the maximum range (mm) of the CoP movement in anterior–posterior and medial–lateral directions. Lower values were considered as indicative of better postural stability. We used the mean values from the three trials for statistical analyses^32^. The CoP data were filtered using a fourth-order zero-lag Butterworth low-pass filter with a 4 Hz cut-off frequency, and processed using custom Matlab routines (R2020a, The Mathworks, Inc., Massachusetts, USA).

#### Walking stability

Stability during walking was expressed by the margin of stability (MoS), and captured via reflective markers on anatomical landmarks^34^ using a 12-camera motion-capture system (Qualysis AB, Gothenburg, Sweden). MoS is based on the inverted pendulum model, where a person is considered stable when the vertical projection of the body centre of mass is kept within the base of support in a static situation. MoS extends the inverted pendulum model of stability in static situations to dynamic situations as it takes into account the velocity of the centre of mass. MoS is defined as the distance between the boundaries of the base of support (BoS) and the extrapolated position of the center of mass (XCoM)^35^, and calculated using Eq. (1). Whole body CoM position was computed based on an average position of four pelvis markers. The XCoM was calculated using Eq. (2).

1 $$ MoS = BoS - XCoM $$

2 $$  XCoM = z + \left( {\frac{x}{{\sqrt {\frac{g}{l}} }}} \right)  $$

Here, *z* is the CoM position, *x* is velocity of the CoM, *g* is the acceleration due to gravity (9.81 m/s^2^), and *l* is an average height of the COM over the whole walking trial^35^. CoM velocity (fore-aft component) was computed by differentiating CoM position with respect to time. The sagittal border of BoS was defined by the anterior–posterior position of the toe marker while the frontal border of BoS was considered as the medio-lateral position of the calcaneal marker. We calculated the anterior–posterior and medio-lateral margin of stability of each step at heel contact. Mean MoS was calculated as the average of instantaneous MoS values for all the participant’s steps (left and right) in each footwear condition. All data were time normalized to 100% of the gait cycle (from initial heel contact to initial heel contact). 3-D motion data was low-pass filtered at 20 Hz using a fourth-order Butterworth filter and processed using custom Matlab routines (R2020a, The Mathworks, Inc., Massachusetts, USA).

#### Mobility

Mobility was assessed with the Timed Up and Go (TUG) test and the Star Excursion Balance (SEB) test. The TUG test is typically used to evaluate basic physical function in older people^36^. The TUG test was timed with a stopwatch and reported in seconds. A longer time to complete the test was considered as worse mobility. Intra-tester and inter-tester reliability for the TUG test have been reported as high in older people^37^. The SEB test is a common measure to assess physical performance in regards to postural-control deficits^38^. Test–retest reliability estimates (ICCs) of the SEB test ranged from 0.91 to 0.95 in a geriatric population^39^ The person performing the test must maintain their stability on one leg, while using the other leg to reach as far as possible in different directions. We employed simplified version of the test where a person reaches out in four directions (anterior, posterior, medial, lateral), instead of eight in the original method. The test was assessed with a measure tape and a mean value from the three trials, in centimetres, was used for the statistical analyses. Shorter reach distance was considered as indicative of worse mobility.

#### Perceptions of shoes

Perceptions of the shoes were assessed using the Monitor Orthopaedic Shoes questionnaire^40^ and scored on a 100-mm visual analogue scale (VAS). The selected questions were related to: (1) attractiveness (0 mm—extremely unattractive, 100 mm—extremely attractive); (2) attractiveness for others (0 mm—extremely unattractive, 100 mm—extremely attractive); (3) comfort (0 mm—extremely uncomfortable, 100 mm—extremely comfortable); (4) fit (0 mm – worst fit possible, 100 mm—best fit possible); (5) ease of donning and doffing (0 mm—extremely difficult to put on and off, 100 mm—extremely easy to put on and off); (6) weight (0 mm—extremely light, 100 mm—extremely heavy), and (7) stability (0 mm—extremely unstable, 100 mm—extremely stable). The questionnaire has been previously validated and is reported to be a reliable measure of perceptions of footwear^40,41^.

### Protocol

Participants completed all assessments in two blocks (Supplementary Figure S2). In the first block, we evaluated participants’ postural stability (posturography) and mobility (TUG test and SEB test) in all three footwear conditions. In the second block, participants completed walking trials in all three footwear conditions to evaluate walking stability and perceptions of the shoes.

#### First block

Following recording of participants’ physical characteristics (age, sex, weight, height), participants were fitted with the first footwear condition and commenced the assessment of postural stability. Participants were required to stand still with both feet on the pressure plate for 30 s. Each test was run three times both with eyes open and eyes closed. Explicit instruction was given to participants i.e. “When I say ‘go’ I want you to close your eyes and to stand as still as possible until you hear the instruction to rest. Keep your arms relaxed by your sides but do not rest your hands on your body”^32^. A test was invalidated and repeated if the participant: (1) changed their foot starting position; (2) changed their arm starting position or (3) opened their eyes during the eyes closed condition.

Next, participants completed the TUG test. Participants began the test sitting upright in a chair with arm rests. The chair was stable and positioned such that it did not move when the participant moved from sit to stand. The participants were allowed to use the arm rests during the sit-stand and stand-sit movements. Explicit instruction was given to participants i.e.: “On the word ‘go’ you will stand up, walk to the line on the floor, turn around and walk back to the chair and sit down. Walk as fast as you can” Timing started on the word “go” and stopped when the participant was seated again correctly in the chair with their back resting on the back of the chair.

Following the TUG test, participants completed the SEB test, which was performed with the participant standing on their dominant leg at the centre of four grid lines drawn on the floor angled at 90 degrees to each other. The four lines were labelled anterior, lateral, medial, and posterior. Participants were instructed to reach with the opposite leg as far as possible in the specified direction while maintaining balance on the stance leg. The maximal reach distance for each trial was recorded from where the toe tapped on a tape. A test was invalidated and repeated if the participant: (1) lost balance, (2) changed their starting foot position; or (3) used the reaching leg for considerable support. On a completion of the SEB test, participants had a 5 min break during which: (1) they were seated, allowing them to rest to prevent fatigue, and (2) they were asked to become accustomed to the new footwear condition by performing a ten-metre walk ten times^28^. After the break they were fitted with another type of footwear and repeated all assessments again.

#### Second block

Once a participant had completed all assessments from the first block in each of the footwear conditions, the second block of tests started. Participants were instructed to perform two tasks over-ground: (1) normal walking, and (2) dual task walking across a 10-m walkway. Dual task walking involved simultaneous walking and counting backwards from a random number ranging from 50 to 200. All trials were conducted at the participant’s self-selected walking speed, and participants performed six to eight trials of each task. During the walking trials, 3D movement of the lower legs, pelvis and trunk were captured via markers on anatomical landmarks^34^ at 200 Hz using a 12-camera motion-capture system (Oqus-7, Qualysis AB, Gothenburg, Sweden). The following locations were used for the markers: hallux, 1st and 5th metatarsal head, calcaneus, medial and lateral malleoli, head of the fibula, medial and lateral epicondyles, greater trochanter, anterior superior iliac spine, xyphoid process, jugular notch; 7th cervical vertebrae. Marker clusters were secured to the head, thorax, sacrum, thighs and shanks using nylon/lycra bands. On a completion of walking trials, we asked participants to fill in the questionnaire related to the perceptions of footwear (except barefoot). Next, participants had an up to 5 min break, after which they were fitted with another type of footwear and repeated the assessments again. Participants were asked to become accustomed to the new footwear condition by performing a 10 m walk ten times^28^.

### Statistical analyses

Data were inspected both descriptively and graphically, and the normality was tested. Numbers (percentages) were used for categorical variables and means (SD) for continuous variables. The distribution of CoP data (postural stability) was skewed; therefore, a natural log transformation was applied to achieve normality, and all statistical analyses for this outcome used the transformed data^42^. A linear mixed effect model was implemented to examine the main effects of footwear (conventional vs. minimal . barefoot) and visual conditions (eyes open vs. eyes closed) on postural stability. An interaction term was introduced to determine whether the effect of footwear type was altered by the visual inputs (i.e. eyes open or closed). The same model was applied to examine the main effects of footwear (conventional vs. minimal vs. barefoot) and walking conditions (normal vs. dual-task walking) on walking stability. The model examining the effects on MoS AP was adjusted for speed and cadence. The model examining the effects on MoS ML was adjusted for speed. We also introduced an interaction terms to determine whether the effect of footwear type was altered in the presence of cognitive load (dual-task walking). We also applied a linear mixed model to examine the main effects of footwear on mobility. The linear mixed effect models employed a first-order autoregressive covariance type and a random intercept was included to allow for variability across individuals (i.e., participant was treated as a random effect). Where appropriate, significant main effects of footwear were followed up with pairwise post-hoc comparisons with Bonferroni corrections. Finally, we used paired-samples t-test to compare the effects of shoes (conventional vs. minimal) on perceptions. Statistical significance was accepted at *p* < 0.05 for the main effects. All analyses were performed using SPSS software, version 25.0 (IBM, Armonk, NY, USA).

## Supplementary information

Supplementary Information.

## Data Availability

The datasets generated during and/or analysed during the current study are available from the corresponding author on a reasonable request.
