# Supplementary material for: Minimal shoes improve stability and mobility in persons with a history of falls
Source: Sci Rep. 2020 Dec 10;10:21755. doi: 10.1038/s41598-020-78862-6 (PMC7730448; doi:10.1038/s41598-020-78862-6)
Supplement: Supplementary file 1 — Supplementary Information. [file 41598_2020_78862_MOESM1_ESM.pdf]

Minimal shoes improve stability and mobility in persons with a history of falls.

Tomasz Cudejko [1], James Gardiner [1], Asangaedem Akpan [1,2], Kristiaan D’Août [1]

[1] Department of Musculoskeletal and Ageing Science, Institute of Life Course and Medical Sciences, University of Liverpool, Liverpool, United Kingdom;

[2] Department of Medicine for Older People & Stroke, Liverpool University Hospitals NHS Foundation Trust, Liverpool, United Kingdom

Supplementary Table S1. Mean values (SD) for the CoP metrics of postural stability according to the footwear and visual conditions (n=30)

	**Conventional**	**Minimal**	**Barefoot**
Eyes open			
AP velocity (mm/s)	13.4 (4.7)	9.7 (2.7)	9.0 (2.4)
AP max range (mm)	3.5 (1.2)	2.7 (1.1)	2.7 (1.0)
ML velocity (mm/s)	9.1 (4.3)	5.6 (1.9)	6.1 (2.4)
ML max range (mm)	2.4 (1.0)	1.6 (0.6)	1.7 (0.7)
Eyes closed			
AP velocity (mm/s)	18.8 (8.4)	13.2 (3.9)	11.9 (3.5)
AP max range (mm)	4.0 (1.6)	3.1 (0.9)	2.9 (1.0)
ML velocity (mm/s)	11.7 (7.1)	6.4 (2.5)	6.3 (3.2)
ML max range (mm)	2.6 (1.4)	1.7 (0.8)	1.6 (1.0)

Lower values are indicative of better postural stability; Abbreviations: AP – anterior-posterior, ML – medial-lateral; mm/s – millimetres/second; max – maximum;

Supplementary Table S2. Mean values (SD) for the spatio-temporal parameters according to the footwear and walking conditions (n=28)

	**Conventional**	**Minimal**	**Barefoot**
Normal walking			
Stance time (s)	0.53 (0.03)	0.54 (0.03)	0.54 (0.05)
Swing time (s)	0.46 (0.03)	0.45 (0.02)	0.44 (0.03)
Stride length (m)	1.44 (0.10)	1.40 (0.09)	1.35 (0.10)
Step length (m)	0.72 (0.04)	0.70 (0.03)	0.68 (0.04)
Cadence (steps/min.)	83.1 (5.68)	85.2 (4.87)	88.8 (5.68)
Dual-task walking			
Stance time (s)	0.54 (0.06)	0.56 (0.06)	0.55 (0.08)
Swing time (s)	0.47 (0.03)	0.44 (0.03)	0.44 (0.03)
Stride length (m)	1.43 (0.10)	1.37 (0.10)	1.32 (0.10)
Step length (m)	0.71 (0.04)	0.69 (0.04)	0.66 (0.04)
Cadence (steps/min.)	84.1 (6.10)	86.5 (6.30)	91.8 (5.95)

Abbreviations: s – seconds; m – meters, steps/min.– steps per minute

Supplementary Table S3. Means (SDs) for the TUG test (n=30) and the SEB test (n=25) according to the footwear

	**SEBT (cm)**				**TUGT (sec)**
	Anterior	***Posterior***	***Lateral***	***Medial***	
Conventional	36.6 (8.3)	26.6 (10.0)	49.8 (9.1)	30.7 (10.7)	7.92 (1.41)
Minimal	37.8 (7.1)	30.8 (9.8)	52.4 (8.4)	34.2 (10.0)	7.62 (1.26)
Barefoot	36.7 (7.0)	26.5 (9.1)	49.6 (9.5)	32.2 (9.5)	7.96 (1.26)

Higher values of the SEB test and lower values of the TUG test are indicative of better mobility; Abbreviations: SEBT – Star Excursion Balance test, TUGT – Timed up and Go test;

Supplementary Table S4. Means (SDs) for the perceptions according to the shoes (n=30)

**Outcome**	**Conventional**	**Minimal**
Attractiveness (mm)	53.2 (29.7)	60.2 (21.6)
Attractiveness for others (mm)	51.9 (26.1)	54.9 (21.5)
Comfort (mm)	71.2 (24.3)	71.0 (23.0)
Fit (mm)	68.7 (24.8)	84.8 (10.1) [1]
Ease of donning and doffing (mm)	65.6 (27.9)	68.0 (23.5)
Weight (mm)	19.4 (14.6)	10.3 (7.2) [1]
Stability (mm)	65.4 (27.6)	86.8 (9.4) [1]

Values are in millimetres on 100-mm visual analogue scales. Higher scores represent greater perceived attractiveness, attractiveness for others, comfort, fit, ease of donning and doffing, weight and stability; [1] Significantly different from conventional shoes;

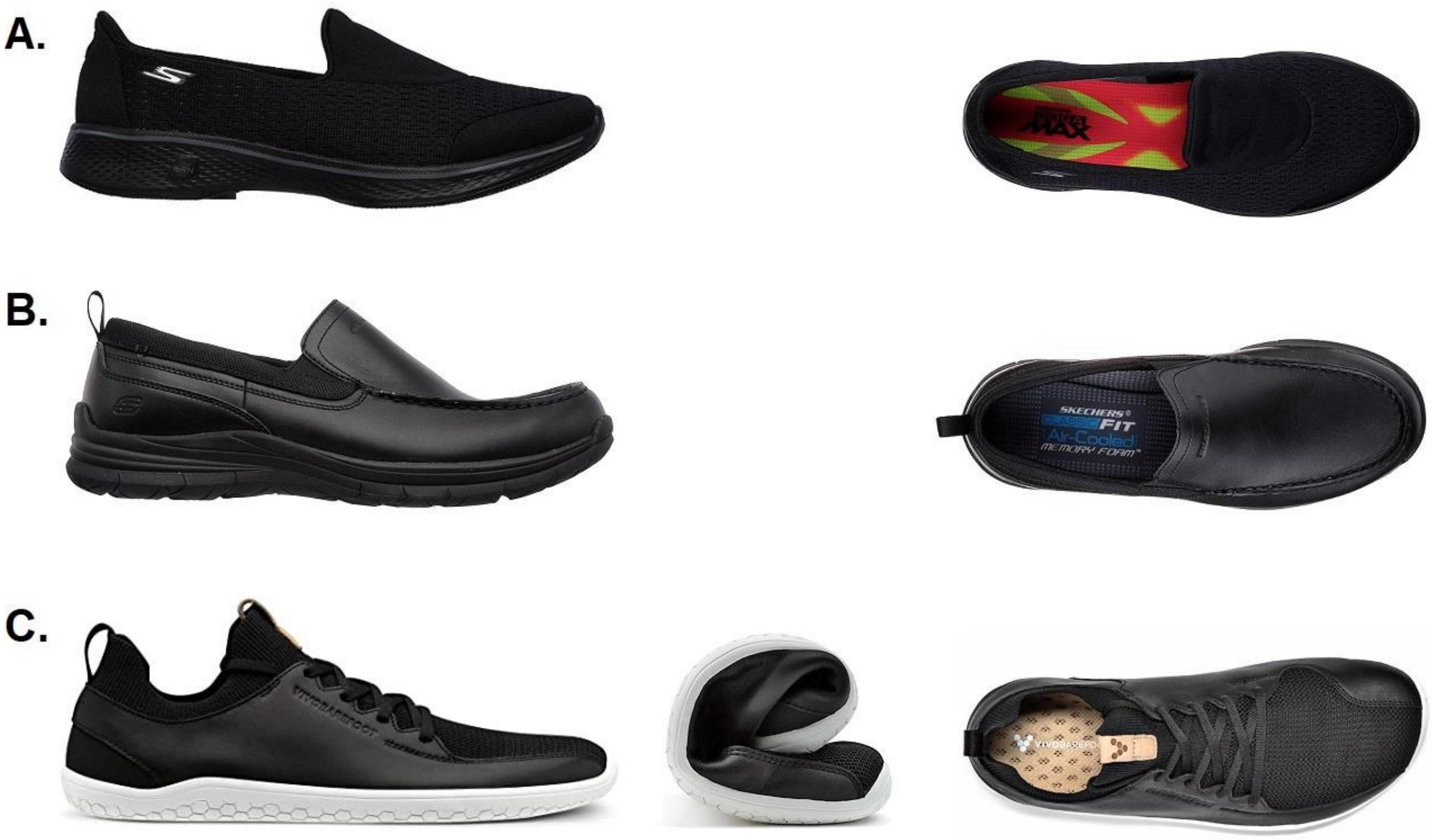

Supplementary Figure S1. A: a female conventional shoe (Go Walk 4.0-Pursuit, Skechers USA, Inc.); B: a male conventional shoe (Superior 2.0-Jeveno, Skechers USA, Inc.); C: a minimal shoe (Primus Knit, Vivobarefoot Ltd., London, UK).

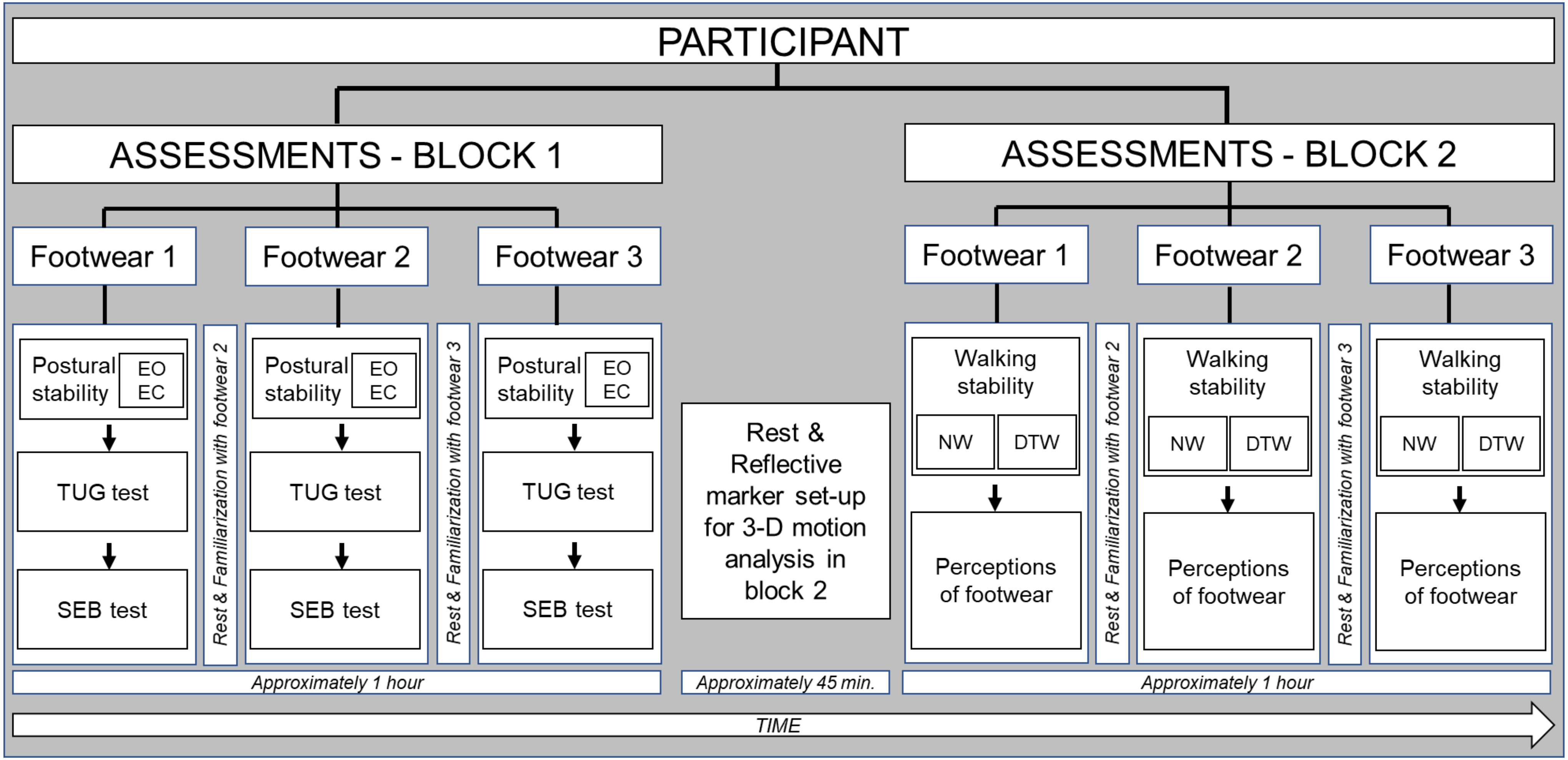

Supplementary Figure S2. Schematic overview of the study protocol per participant; Abbreviations: EO – Eyes open, EC – Eyes closed, TUG – Timed Up and Go test, SEB – Star Excursion Balance test, 3-D – Three-dimensional, NW – Normal walking, DTW – Dual-task walking
